# Practitioners’ views on community implementation of point-of-care ultrasound (POCUS) in the UK: a qualitative interview study

**DOI:** 10.1186/s12913-023-09069-4

**Published:** 2023-01-25

**Authors:** Joseph N.A Akanuwe, Aloysius Niroshan Siriwardena, Luc Bidaut, Pauline Mitchell, Paul Bird, Daniel Lasserson, Patricia Apenteng, Richard Lilford

**Affiliations:** 1 Community and Health Research Unit, School of Health and Social Care, University of Lincoln, Lincoln, England; 2 School of Computer Science, College of Science, University of Lincoln, Lincoln, England; 3 School of Health and Social Care, University of Lincoln, Lincoln, England; 4 Institute for Translational Medicine Research & Development, University Hospitals Birmingham NHS Foundation Trust, West Midlands Academic Health Science Network, Birmingham, England; 5 Warwick Medical School, University of Warwick, Coventry, England; 6 Department of Gerontology, Oxford University Hospitals NHS Foundation Trust, Oxford, England; 7 Institute of Applied Health Research, University of Birmingham, Birmingham, England

**Keywords:** Point of care ultrasound, Community practice, Primary care, Prehospital care, Ultrasound practitioners, Facilitators

## Abstract

**Background:**

Implementing Point-of-care ultrasound (POCUS) in community practice could help to decide upon and prioritise initial treatment, procedures and appropriate specialist referral or conveyance to hospital. A recent literature review suggests that image quality, portability and cost of ultrasound devices are all improving with widening indications for community POCUS, but evidence about community POCUS use is needed in the UK. We aimed to explore views of clinical practitioners, actively using ultrasound, on their experiences of using POCUS and potential facilitators and barriers to its wider implementation in community settings in the UK.

**Methods:**

We conducted a qualitative interview study with practitioners from community and secondary care settings actively using POCUS in practice. A convenience sample of eligible participants from different clinical specialties and settings was recruited using social media adverts, through websites of relevant research groups and snowball sampling. Individual semi-structured interviews were conducted online using Microsoft Teams. These were recorded, transcribed verbatim, and analysed using a Framework approach supported by NVivo 12.

**Results:**

We interviewed 16 practitioners aged between 40 and 62 years from different professional backgrounds, including paramedics, emergency physicians, general practitioners, and allied health professionals.

Participants identified key considerations and facilitators for wider implementation of POCUS in community settings in the UK: resource requirements for deployment and support of working devices; sufficient time and a skilled workforce; attention to training, education and support needs; ensuring proper governance, guidelines and quality assurance; workforce considerations; enabling ease of use in assisting decision making with consideration of unintended consequences; and more robust evidence to support perceptions of improved patient outcomes and experience.

**Conclusions:**

POCUS could be useful for improving patient journey and health outcomes in community care, but this requires further research to evaluate outcomes. The facilitators identified could help make community POCUS a reality.

**Supplementary Information:**

The online version contains supplementary material available at 10.1186/s12913-023-09069-4.

## Background

In the wake of the current pandemic, the Beneficial Changes Network (BCN) was established in England to explore how health care provider systems and the public used innovations to respond to COVID-19 and to continue to provide quality healthcare for individual patients and populations. In line with this, point-of-care ultrasound (POCUS) has been identified as technology that could support clinical practice in community settings, particularly in prehospital and primary care.

POCUS involves the use of a highly portable ultrasound system at the side of the patient wherever they might be. While this might be by trained clinicians at the patient’s bedside in hospital settings [1], the increasing ubiquity and portability of such devices mean they might be used especially in pre-hospital settings and by users other than fully trained bona-fide medical imagers. Accordingly, many such devices are now often complemented by digital assistants to help with both acquisition and some interpretation of the images ahead of further workup [2]. These now include ‘hand-held’ devices which have been described as ‘modern stethoscopes’ because they are portable and can be used in a range of clinical environments [3].

POCUS could play an important role in patient care, as evidence suggests POCUS could help to prioritise initial treatment and transportation of a patient to the most appropriate hospital [4, 5]. This is supported by one systematic review [6] which suggested that prehospital POCUS may improve patient management in terms of diagnosis, treatment and hospital referral, although this review also suggested there was a high risk of study bias, and heterogeneity between studies made further analysis impossible [6].

A further systematic review found that image quality and portability of ultrasound devices were improving while equipment costs were decreasing, and indications for prehospital POCUS were widening [7]. Another review found POCUS was increasingly being used by General Practitioners (GPs) and Emergency Practitioners (EPs) in many countries, and that generalists could safely use ultrasound in a range of clinical settings to aid diagnosis [8]. A recent study, which explored GPs’ use of POCUS and its influence on the diagnostic process and treatment of patients, found POCUS examinations in general practice were used for many different indications, providing diagnostic reassurance for the GP and a change in diagnosis or management in 71.8% of patients  [9].

Despite these positive developments, most of the evidence comes from outside the UK, and there is therefore a need for further research about the use or role of POCUS in the UK to support its future implementation in UK community settings [8, 10]. This study aimed to explore the views of clinical practitioners, actively using ultrasound, on their experiences of using POCUS and potential facilitators and barriers to its wider implementation in UK community settings.

## Methods

### Design and setting

We conducted a qualitative interview study with practitioners who used POCUS in their routine practice. Participants were from community and secondary care settings, and interviews were conducted online via Microsoft Teams.

### Theory

We used non-adoption, abandonment, scale-up, spread, and sustainability (NASSS) technology implementation framework [11] and Diffusion of Innovation theory [12] to inform data collection and analysis.

### Sampling and sample size

A convenience sampling approach through social media and a snowballing technique were used to recruit eligible participants. The inclusion criteria were practitioners (irrespective of gender) using ultrasound in practice, willing to participate in individual interviews using telephone or video, and willing to give informed consent. The research team had expertise in qualitative research. It was estimated that 15 to 20 participants would generate sufficient data to address the objectives of the study, but we planned to continue interviewing until data saturation in code and meaning [13] was achieved.

### Recruitment and data collection

Following ethics approval, participants were identified by using a flyer advertising the study on social media (Twitter) and websites of relevant research and professional groups. This was supplemented using a snowballing approach with those interviewed identifying further participants. Interested participants contacted the researcher (JA) who sent further details of the study, including a Participant Information Sheet and a Consent Form, by email.

Interviews were conducted via Microsoft Teams (video conferencing software), each lasting around an hour, following a topic guide that had been collectively developed by the research team (see below).

### Data analysis

The interviews were audio recorded, transcribed verbatim and analysed using a Framework [14] approach supported by NVivo 12 software (QSR International). An initial framework of codes was based on the domains of the non-adoption, abandonment, scale-up, spread, and sustainability (NASSS) technology implementation framework [11] that informed the interview schedule. Building on Diffusion of Innovation theory [12] and its updated version, [15] the NASSS framework was developed to help generate ideas towards the implementation of innovative technology [11]. Hence, the perceived facilitators to the implementation of POCUS were considered in the context of seven domains within the framework: the condition; the technology; the value proposition; the adopter system (staff, patient, and lay caregiver); the healthcare organisation involved in the implementation process; the wider institutional and societal context; and the interactions and adaptations over time [11].

With the initial codes in mind, the various stages of Framework analysis were followed: familiarisation; identifying a thematic framework; indexing; charting; mapping and interpretation [14]. Subsequently, similar codes were grouped and regrouped to form higher order themes and sub-themes.

This systematic approach provided a clear audit trail from raw data to final themes, thus ensuring trustworthiness of the results [16, 17]. In addition, an open and reflexive approach ensured a rigorous qualitative data analysis [18], and the Consolidated Criteria for Reporting Qualitative Studies, as shown in Supplementary Table S1 [19], was also followed, thus adding further trustworthiness to the results.

## Results

A total of 16 practitioners, aged between 40 and 62 years, with a range of professional backgrounds including paramedics, prehospital emergency physicians, general practitioners (GPs) and allied health professionals, participated in the interviews (Table 1 below).

**Table 1:** Participant characteristics

ID	Gender	Age (years)	Ethnicity	Professional background	Time in role (years)	Setting
1	Male	36–40	African	Emergency medicine registrar/physician	1–5	Secondary care (emergency department/critical care)
2	Female	41–45	Asian British (Indian British)	Acute/emergency medicine consultant	1–5	Acute (hospital) and community (general practice)
3	Male	41–45	White British	GP—private practice	1–5	Community (General practice, hospital)
4	Male	31–35	White British	Emergency medicine registrar/physician	1–5	Acute (hospital) and community (home)
5	Male	51–55	Asian British (Indian British)	Emergency medicine registrar/physician	1–5	Secondary care (emergency department/critical care
6	Male	41–45	Asian British (Indian British)	GP	16–20	Secondary care (emergency department/critical care)
7	Male	61–65	White British	Allied health professional (Radiologist, sonographer, Physiotherapist)	26–30	Secondary care -emergency department, critical care
8	Male	36–40	Arabic	Acute/emergency medicine consultant	1–5	Secondary care (emergency department/critical care)
9	Male	51–55	White British	Acute/emergency medicine consultant	11–15	Secondary care (emergency department/critical care)
10	Male	41–45	White British	Allied health professional (Radiologist, sonographer, Physiotherapist)	1–5	Community (General practice, hospital)
11	Male	31–35	Pakistani	Emergency medicine registrar/physician	6–10	Acute (hospital) and prehospital
12	Male	31–35	White Australian	Paramedic (intensive/critical care)	1–5	Community emergency care/prehospital
13	Male	51–55	White British	Paramedic (intensive/critical care)	31–35	Community emergency—prehospital
14	Male	36–40	White British	Paramedic (intensive/critical care)	1–5	Community emergency—prehospital
15	Male	36–40	White British	Prehospital/hospital emergency physician	6–10	Community emergency—prehospital
16	Male	41–45	White British	Paramedic (intensive care, critical care)	6–10	Community emergency—prehospital

**Fig. 1:**
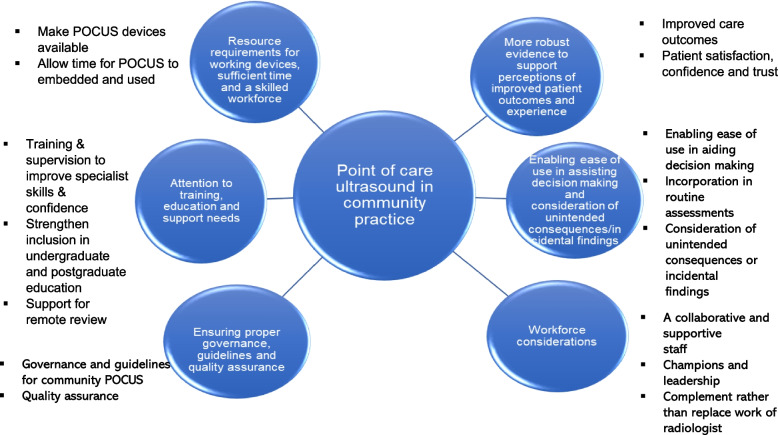
Thematic map

Participants agreed that community POCUS could be useful but was not currently widely available in community settings in the UK. We identified several factors organised into themes and subthemes, perceived by interviewees to facilitate wider implementation of POCUS in community settings in the UK (Fig. 1 below).

### Resource requirements for working devices, sufficient time and a skilled workforce

#### Make POCUS devices available

Participants felt community health care organisations should invest in portable and well-functioning ultrasound machines and ensure these are available for use by practitioners:

*“So, if you’re working on that, the very first thing to solve now—the issue you can solve more easily than any other thing—is the availability of machines. So, make pocket-sized [brand X] machines more available and see how this affects practice. I think that’ll be the best thing to do”: *Practitioner 1.

*“I think the first thing would be to invest in the equipment”: *Practitioner 3.

*“There needs to be financial backing to buy the machines because they’re definitely not cheap”:* Practitioner 10.

#### Allow time for POCUS to be embedded and used

Participants viewed POCUS as a technology that was “*just a recent thing within the last five to ten years”:* Practitioner 3. Participants also felt that, in addition to making the machines available, it will be beneficial to community practice if they could allow extra time for POCUS:

*“I don’t think there is any interaction in primary care that wouldn’t benefit from giving a little bit more time to doing it”:* Practitioner 4

*“They should have time in their job plans so that this isn’t just an added extra that people do off their own back out of kindness and civic duty. They should be recognised and have time to do it”: Practitioner 4.*

### Attention to training, education and support needs

#### Training & supervision to improve specialist skills & confidence

Practitioners identified that a commitment to training and supervision over time was needed to enhance specialist skills and confidence in wider community use of POCUS:

*“There are clearly training issues because if you want to train a doctor, unless he’s a radiologist, then he does need to do a training course, and depending on the extent or the sort of condition that you wish to use [POCUS] for, that training period could be anywhere from two months to a year. So, there is a training commitment to it”: *Practitioner5.

*“So, the point is that there needs to be support in order to gain accreditation or the necessary courses”: *Practitioner10.

#### Strengthen inclusion in undergraduate and postgraduate education

It was suggested by participants that some medical schools in the UK were beginning to include POCUS education in their curriculum, so that doctors and other clinicians could be equipped with awareness, knowledge and skills on POCUS as part of their training. It was felt that the inclusion of POCUS in medical education needed to be strengthened across undergraduate and postgraduate medical programmes in the UK:

*“We need to start putting POCUS in medical schools’ hands”: *Practitioner 3.

*“The fact that it’s now becoming commonplace in undergraduate education is important because you’re going to have a generation of doctors that have started earlier. So, it’s not going to be the case that your first experience with POCUS is when you’re already a practicing doctor. Your practice will have started earlier, so it’ll just be seen as routine and commonplace”: *Practitioner 4.

To strengthen the knowledge and skills gained from undergraduate training, postgraduate medical education should include POCUS in the training pathways of doctors, paramedics and other clinicians who could potentially implement POCUS in community practice:

*“Royal colleges also need to make this part of a training pathway for doctors, and for some specialities that’s already in place. If you think about intensive care, doctors training in intensive care have to undertake ultrasound training, and the same is true for emergency medicine. But for community specialities such as general practice or doctors that are working within an urgent care setting for example, or even for pre-hospital emergency medicine training, ultrasound is not within those curricula. So, for this to become a reality, we need to invest or get those organisations on board”: *Practitioner 15.

#### Support for remote review

Support for remote review was perceived as potentially useful for community practice, particularly for situations such as road traffic accidents in a prehospital setting, or when a GP required a more qualified or experienced clinician to remotely guide or interpret the POCUS results.

*“… if an ambulance goes to a road traffic accident and they think the patient has fluid in the abdomen, then the ambulance paramedic could do an ultrasound and there could be somebody in secondary care who could do remote access to help that paramedic”: *Practitioner 7.

*“So, if a practitioner does a scan in primary care, then in the future, there will hopefully be superusers in a secondary care setting—whether that’s in the military or the non-military—and the remote access will allow the scan to be guided. So, say a GP puts a probe on somebody’s abdomen and they’re unsure of what they’re seeing and how to do the next step, then, in the future, they could contact somebody in secondary care using remote access, because several ultrasound manufacturers have this facility now”:* Practitioner 7.

As the technology continues to improve, it was felt that remote reviewing would become easier:

*“But equally, the technology is kind of getting to the point where that’s going to become easier because the [POCUS machines of brand X] now have a cloud-based system where I can review scans in real-time. So, someone can upload them, and I’ll get an email and I can review the scans. There’s even a telemedicine function. One of my colleagues that I know from work has actually done a live telemedicine session. He works in the army as well, and he’s actually done some live training where someone’s doing an echo and he’s basically just on a computer back home and he’s telling them how to move the probe, how to get the views and stuff like that. So, there is technology coming on board that’ll make training easier to do remotely”:* Practitioner 9.

### Ensuring proper governance, guidelines and quality assurance

#### Governance and guidelines for community POCUS

Concerns about lack of governance and guidelines for community POCUS were expressed by participants who felt that these would increase trust in the technology from other professionals:

*“Certainly, for other professionals to trust you, there has to be good governance in place”: *Practitioner 10.

Participants suggested that robust governance systems, that currently exist in secondary care should be replicated for implementation of community POCUS:

*“So, what we’ll do is use the same governance protocol that we use for secondary care, where you attend a course, you do a logbook, you do your CPD, you have a mentor, and then you have quality assurance and regular audits”: *Practitioner 2.

#### Quality assurance

Participants felt that quality assurance could be achieved by establishing and maintaining competence and confidence in community POCUS:

*“Secondly, any scanning that you do, obviously you have to make sure you’re competent and there’s quality assurance”:* Practitioner 2.

### Workforce considerations

#### Collaborative and supportive staff

Clinicians with more knowledge and experience of POCUS could contribute to the deployment and implementation of POCUS in community care settings by collaborating and offering support to less experienced colleagues:

*“Yeah, so already within my practice, a lot of my colleagues send patients to me to assess patients with POCUS. So yeah, when they have a question that they want to ask me, they’ll send them through to me. Some colleagues use me just for ultrasound-guided procedures”: *Practitioner 3.

The support offered by more experienced clinicians may include sharing knowledge and encouraging others to appreciate the benefits of POCUS:

*“I know how useful it is, it’s just about trying to impart that knowledge and enthuse other people towards it. That’s not that easy, but I do have people that now realise how good the service is, and I’m starting to develop it more”: *Practitioner 10.

#### Champions and leadership

Participants suggested that identifying and establishing POCUS champions and leaders could help to drive community implementation:

*“So, the plan would be to have some champions within GPs to prove concept, and then you start the momentum. And we already have some champions who are already doing it, the ones that I’m mentoring, so there are quite a few — there are a few, there are about four or five in the UK, so there’s a long way to go”: *Practitioner 2.

#### Complement rather than replace work of radiologists

Another workforce issue that needed to be considered was the potential risk of perceiving POCUS as replacing the work of radiologists. Participants made clear that use of community POCUS should complement the work of radiologists rather than replacing them:

*“I think what people need to understand is that POCUS is definitely not replacing department hospital radiology ultrasound. POCUS is for a completely different situation. You’re using it to aid your patient assessment at the bedside or in your surgery”: *Practitioner 6.

*“I can’t say for a second that I’m as good with a stethoscope as a cardiologist would be at diagnosing the nature of that murmur I’m listening to, but I can tell you that there’s a systolic or a diastolic murmur and I can tell you it’s loudest over the aortic or the mitral region. And that’s the same with POCUS. I wouldn’t dream for a second that I would replace the role of a highly experienced radiologist”: *Practitioner 3.

### Enabling ease of use and aid to decision making versus consideration of unintended consequences or incidental findings

#### Enabling ease of use in aiding decision making

Participants welcomed devices becoming small, portable and more efficient, enabling their use and enhancing decision making:

*“It’s not just [machine X], there’s loads, such as [machine Y] and [machine Z]. The advantage is its portability: you can put it in your bag, take it out to wherever you need to, and you can make a decision”: *Practitioner 2

*“But then I have to pinch myself and say, “Hang on a second; I’ve got something that fits in my pocket, that plugs into my phone, and that I can use within ten seconds”: *Practitioner 3.

#### Incorporation in routine assessments

Participants felt POCUS could be incorporated in routine community assessment, as an extension of the clinical examination:

*“It’s just become an extension of clinical examination. I take a history, I examine the patient with my hands and use my stethoscope, and then I get the ultrasound out and do a focused ultrasound examination. I do that pretty much for every patient”: *Practitioner 4.

#### Consideration of unintended consequences or incidental findings

Unintended consequences or unintended findings needed to be considered. For example, incorrect use of the device or interpretation of the results could lead to an incorrect assessment or care pathway, which might not benefit the patient:

*“So, practitioners who have no education in ultrasound may use it incorrectly—in terms of either diagnosing or not diagnosing disease—and they may not have the correct communication or patient pathway, which therefore means the patient doesn’t benefit”: *Practitioner 7*.*

*“On the flip side, if you don’t do a full examination, POCUS can lead to a false diagnosis, or it can lead you to another path even though the patient might not have this problem. To give you an example from my own experience, I had a patient come in who was really short of breath, really tachycardic and had a raised JVP. I think my examination of her chest was a bit limited, as I didn’t realise that she didn’t have reduced breath sounds on the left side, so I did an echo and thought that she had a tamponade. But she didn’t have a tamponade; she had something called a tension hydrothorax. So, she had a massive pleural effusion that was causing compression of her heart. So, I think it’s about being mindful of how we use ultrasound”: *Practitioner 11.

### More robust evidence to support perceptions of improved patient outcomes and experience

#### Improved care outcomes

Some participants felt that while the use of community POCUS may not directly lead to improvement in patient health outcomes, it could enhance patient assessments leading to more accurate diagnosis and treatment, which could prevent a patient’s condition from getting worse:

*“Using POCUS in and of itself isn’t going to prevent a patient from getting worse in the out-of-hospital setting, but the ability to gain IV access and being able to deliver inotropes and fluids where previously you wouldn’t, could potentially prevent a patient’s condition from worsening. Preventing a patient with heart failure from getting 10 mg of salbutamol nebulised could prevent them from getting worse. I can’t think of too much else off the top of my head”: *Practitioner 12.

#### Patient satisfaction, confidence and trust

Patient satisfaction, alongside confidence and trust, was viewed by participants as a potentially important care outcome. Some participants felt patients were likely to feel more satisfied, having confidence and trust in a clinician who could assess their illness by scanning them at the point of care:

*“I haven’t come across any patient who has shown me dissatisfaction, because what patients want when they go and see a doctor is to find out what’s wrong with them. So, if you can say to them, “Actually, I’ve scanned you; here are gallstones that are causing your problem,” patients are immensely grateful because that’s what they came for”:* Practitioner 5.

Furthermore, POCUS can allow a patient to see how they are being examined, adding more patient satisfaction and trust in the service provided:

*“Yeah, when I turn on the doppler ultrasound and they can hear their valve working and the sound of their heart, the patient looks more satisfied with the service. They trust more. They know that I’m really seeing their heart and really examining them. I’m not just fooling around with the ultrasound. I think they trust in our service”:* Practitioner 1.

While the views expressed suggests that POCUS could lead to some improved patient care outcomes, further quantitative evidence to support the impact of POCUS on patient health outcomes and experience following implementation in community care settings is needed in the UK.

## Discussion

### Main findings

Key facilitators for implementing POCUS in community settings included resource requirements for working devices, sufficient time and a skilled workforce; attention to training, education and support needs; ensuring proper governance, guidelines and quality assurance; workforce considerations; enabling ease of use in assisting decision making and consideration of unintended consequences; and a need for more robust evidence to support and further qualify perceptions of improved patient outcomes and experience.

Investing in portable and capable ultrasound machines and making these available to practitioners is one important resource requirement. Previous studies suggest that the cost of equipment, training opportunities [20, 21] and fiscal constraints [19] have limited the availability of ultrasound machines. More recent falls in prices of pocket or handheld POCUS equipment [20, 21] suggests that these devices are becoming more affordable for healthcare organisations to make available to community practitioners. This reflects the views of practitioners in our study, who felt that portable POCUS devices should be made more available in the community. Regarding cost and affordability of clinically-competent POCUS devices, the cheapest one on the market to-date is the Butterfly iQ (about £2,500—one off cost, plus £350 per year for image storage) whereas the GE VScan Air is around £6,000. Costs are therefore relatively cheap in context of traditional diagnostic imaging costs (a ‘cart’ ultrasound in hospital is over £30,000 with additional maintenance costs of at least 10% per year). Full pathway analyses for POCUS costs vs. benefits have yet to be undertaken but ruling in or ruling out key conditions in community settings can save on hospital costs and the cost of missing treatable disease. Looking at cost in terms of durability, the technology of piezoelectric crystals generating sound at very high frequency is the same for the majority of probes for both POCUS and conventional ultrasound systems. The actual durability is thus mainly related to how the probes are cared for (e.g. taking care not to drop them).

Allowing time for POCUS to be embedded and used, as any new technology, was also viewed as necessary for effective community implementation of POCUS. Evidence suggests that when POCUS is performed on-scene in the community, a slight delay may occur unless POCUS is performed with other procedures, such as during transport to the most appropriate hospital, which could even save time. [9] This is more so in the UK, where patients need to travel to hospitals for ultrasound examinations. If local primary care practices had their own ultrasound (e.g. POCUS), then related travel would be much shorter (hospitals cover on average 500,000 patients, primary care practices an average of 8,000 each).

Attention to training, strengthening undergraduate and postgraduate education, and support needs such as remote review of POCUS was also considered important for community implementation. While educational programmes have been developed for clinicians working in hospital settings, [22, 23] ultrasound training for community staff such as general practitioners (GPs), is limited [9] and requires strengthening in the UK. Practitioners in this study acknowledged that while POCUS was being introduced into some medical training programmes in the UK, in their view this needs to be strengthened across all undergraduate and postgraduate medical education and training pathways of doctors, paramedics and other clinicians who might use POCUS in community practice. Lessons can be learnt from existing medical school curricula [24, 25] and specialist training programs [23, 26].

Support for remote reviews by a more qualified and experienced ultrasound practitioner or radiographer was perceived as important for enhancing implementation as not all POCUS users will have a broad and deep enough training in image interpretation [27]. As the technology continues to improve, remote reviewing and reporting may become more available and affordable.

Ensuring proper governance, guidelines and quality assurance were identified by participants as a key consideration for effective and safe community POCUS [28, 29]. Existing governance and guidelines are based mainly on secondary care and may not directly apply to community care settings, [30, 31] particularly in the UK but could be modified to suit community practice, taking into consideration scope of practice, integration of findings into clinical management, review of relevant guidelines, policy development and documentation [29].

Quality assurance as part of governance was viewed by participants as achievable if competence and confidence in community POCUS are established and maintained. However, a recent systematic review of POCUS in primary care found that quality depended on the extent of the examination and the anatomic area being scanned, with more focused scans having higher levels of diagnostic accuracy, requiring less training, and being associated with less potential harm, while more extensive examinations were associated with lower scan quality and more potential harm [9].

Clinicians with more knowledge and experience of POCUS could offer support and share knowledge and skills with less experienced colleagues and act as champions to help drive and lead a wider implementation of POCUS, but because of the lack of community use, might require help from secondary care POCUS practitioners. There is risk of people perceiving POCUS as replacing the work of radiologists. Participants felt that users of POCUS in community settings should aim to complement rather than replace the work of radiologists, which aligns with findings from previous studies that POCUS is not the same as ultrasound examinations performed by radiologists or other highly specialised physicians, [32] but that clinicians should be encouraged to apply POCUS as part of physical examination of patients [33, 34]. As the devices have become smaller, more portable, efficient, and easier to use, more practitioners have adopted POCUS as an extension of physical examination and clinical assessment, [27] where it may influence decision-making in prioritising initial treatment and referral or conveyance to hospital [4, 5]. This has to be balanced against the risk of misdiagnosis leading to unnecessary concerns or anxiety for patients or delay in potentially lifesaving treatment if a serious condition is missed [9].

Finally, providing more robust evidence to support improved care outcomes and patient experience could facilitate POCUS implementation. This is supported by evidence suggesting that adding POCUS to clinical examinations can contribute to improved and earlier diagnosis in hospital settings, [35, 36] although further research is needed to evaluate the impact of community POCUS.

## Strengths and limitations

To the best of our knowledge, this is the first study exploring the views of POCUS practitioners to inform the implementation of POCUS in community practice in the UK. Although the study was conducted during the pandemic with many clinicians being very busy, our recruitment approach, through social media supported by snowballing, yielded a sufficient number of practitioners from a range of professional backgrounds who provided in-depth information for the study, enabling data saturation to be achieved [13]. Our findings may not be generalisable outside the UK, because clinical training differs from country to country. For example, in Germany, ultrasound is part of many specialty training pathways and general practitioners are already competent in its use. Single payer systems still have multiple providers with allocated budgets, so one provider purchasing technology that benefits another provider in a care pathway will not provide sufficient motivation unless a clear strategic direction is supported by related research.

Findings, particularly around patient health outcomes and experience, were based on perceptions of participants. Only two primary care practitioners participated in the study. By recruiting participants through social media, it is possible that many respondents already were enthusiastic about POCUS and its usefulness, and other, perhaps more casual, POCUS users with possibly different views might not have engaged as readily as our participants did.

### Implications for practice and research

Implementing POCUS in community practice could help to improve patient experience, care pathways and outcomes. This could be achieved by integrating POCUS in clinical assessments or examinations, which could then lead to more accurate and earlier diagnosis and treatment. The facilitators reported in this study should be considered when planning for wider implementation. A key requirement for implementation is the need for more training of clinicians on POCUS. Further research is needed to explore the extent to which POCUS is being integrated into clinical education and how best this can be strengthened. Further research is also needed to assess the impact of community POCUS on patient outcomes and experience.

## Conclusion

POCUS could be useful for improving community care processes, patient outcomes and experience. Participants identified several facilitators that could support wider implementation of POCUS in community practice in the UK. These will need to be taken into consideration for making POCUS in community practice in the UK a reality.

## Supplementary Information

**Additional file 1:** **Table S1.** Consolidated criteria for reporting qualitative studies (COREQ): 32-item checklist.

## Data Availability

The datasets generated and/or analysed during the current study will be available in the University of Lincoln repository (https://eprints.lincoln.ac.uk/) and will be accessible upon request by contacting the corresponding author (nsiriwardena@lincoln.ac.uk).

## Abbreviations

BCN: Beneficial Changes Network
CF: Consent form
EPs: Emergency Practitioners
GPs: General Practitioners
IV: Intravenous
JVP: Jugular Venous Pressure
NASSS: Non-adoption, Abandonment, Scale-up, Spread, and Sustainability
PIS: Participant Information Sheet
POCUS: Point of Care Ultrasound
UK: United Kingdom
